# Army News

**Published:** 1918-11

**Authors:** 


### Army News.

Dr. R. Y. Lacy, of Pittsburg, Texas, has accepted a captain's commission and is now in the M. R. C. at Camp Greenleaf, Ga.

Dr. A. B. Kennedy of Bonham, Texas, has lately been commissioned major in the medical corps of the army. He will shortly report to Camp Logan, Houston, to assume his duties.

Dr. Grady Shytles, who has been connected with the United States public health service in Fort Worth and who was former assistant city health officer, has been detailed to the Navajo Indian reservation at Flagstaff, Ariz., to take charge of the fight against an influenza epidemic among the Indians.

Dr. David L. Bettison, who has been a member of the faculty of Baylor Medical College for nine years has been commissioned lieutenant in the medical corps. He volunteered his services several months ago. Lieutenant Bettison has gone to Camp MacArthur, where he will be stationed at the base hospital.

Dr. A. Murice Spurgin of Dallas has received a commission as captain in the medical corps and has been assigned for duty at Camp MacArthur.

Dr. W. E. Chilton, of Fort Worth, has received notification from the war department that he has been accepted for service and commissioned a captain in the medical corps.

He was instructed to report to Fort Oglethorpe, Ga.

Dr. E. V. Dickey, of Dickey & Dickey, well-known Dallas physicians, has accepted a first lieutenancy in the hospital service of the government. Dr. Dickey will report for duty at Camp Travis in the near future.

Dr. W. G. Cook, Fort Worth physician, has been commissioned a captain in the United States Army, Medical Reserve Corps. He has been ordered to report for duty the latter part of this month.

His first assignment will be at the Camp Bowie Base hospital.

Capt. Henry M. Osborne, Medical Corps, has been ordered from Camp Bowie to Camp Hancock, Augusta, Ga., where he will report to the base hospital for duty.

Capt. C. C. Smith of the Bowie Base hospital, has gone to Camp Crane, Allenton, Pa., for temporary duty.

Maj. George H. Day of the Camp Bowie Base Hospital has received orders transferring him to Camp Pike, Ark., for temporary duty. Major Day has been at the Bowie Base hospital for nearly a year.

Dr. Leonard F. Bland, of Dallas, recently commissioned a lieutenant, has been ordered to report at Fort Oglethorpe, Ga., to take up special work.

Dr. H. R. Levy, of Dallas, who was born in the Province of Lomza, Poland, at the German border, has been commissioned a first lieutenant in the United States Army Medical Corps. His parents are in Germany now.

Dr. Levy had military experience as a first lieutenant, Medical Corps, Texas National Guard, having been appointed by Governor Colquitt, and he later was promoted to a Captaincy in 1914, resigning in 1916. He received his commission in the United States Army Medical Corps on October 15 and was ordered to report for duty October 30 at Camp Logan, Houston.

Dr. Samuel Ramsey Milliken, of Dallas, has been ordered to report to Camp Greenleaf, Fort Oglethorpe, Ga., for duty.

Dr. Albert Nash, of Dallas, who was recently commissioned a lieutenant in the medical corps, has been ordered to Camp MacArthur.

Dr. G. Graham, of Gonzales, Texas, who is stationed at West Point, Kentucky, has recently receiced his commission as captain.

Dr. Mary Lee Edwards, of the staff of the Women's Overseas Hospital, now in France, has been awarded the French Croix de Guerre. At the same time she received her commission as a lieutenant in the French Army. The hospital of which Dr. Edwards is a member was organized by the Women's Suffrage Party.

Dr. John H. Traylor, a member of the staff of the Burns Hospital at Cuero, for the past several years, is now located at Camp MacArthur, Waco, where he entered the base hospital with the rank of first lieutenant.

Dr. H. G. Walcott of Dallas, who was recently commissioned captain in the medical corps, is now in charge of the base hospital at Camp Travis.

Dr. D. A. Mohler, of Dallas, has received his commission and will soon report for duty. Dr. Mohler has been commissioned a lieutenant in the Aviation Service and ordered to report to Kelly Field at San Antonio.

Dr. J. F. Howze of Austin, Texas, has accepted a commission in the medical department of the United States Army for service abroad.

Dr. W. D. Jones of Dallas, Texas, has been commissioned a captain in the medical reserve corps. He is now located at Camp Fremont, Palo Alto, Cal., where he is stationed at the base hospital and assigned to the eye, ear, nose and throat service.

Dr. F. M. Hale, of Ballinger, who volunteered for service in the medical corps of the army, and who was assigned to duty in the army camp at Oglethorpe, Ga., has been transferred to a training camp for overseas duty.

Dr. H. I. Warwick, of Fort Worth, who recently received a commission as captain in the medical corps of the United States Army, has received notification to report at Camp Logan.

Dr. Warwick is a specialist on the ear, eye, nose and throat and will continue his work along this line when he goes into the service of the army.

Capt. I. A. Withers, Fort Worth physician, who volunteered and was sent to Fort Oglethorpe some time ago, has been transferred to a base hospital in Alabama.

